# Conceptualizing and communicating management effects on forest water quality

**DOI:** 10.1007/s13280-015-0753-6

**Published:** 2016-01-07

**Authors:** Martyn N. Futter, Lars Högbom, Salar Valinia, Ryan A. Sponseller, Hjalmar Laudon

**Affiliations:** Department of Aquatic Sciences and Assessment, Swedish University of Agricultural Sciences, 750 07 Uppsala, Sweden; Skogforsk, Uppsala Science Park, 751 83 Uppsala, Sweden; Norwegian Institute for Water Research, Gaustadalléen 21, 0349 Oslo, Norway; Department of Ecology and Environmental Science, Umeå University, 901 87 Umeå, Sweden; Department of Forest Ecology and Management, SLU, Skogsmarksgränd, 901 83 Umeå, Sweden

**Keywords:** Boreal, Environmental communication, Forestry, Water quality

## Abstract

We present a framework for evaluating and communicating effects of human activity on water quality in managed forests. The framework is based on the following processes: atmospheric deposition, weathering, accumulation, recirculation and flux. Impairments to water quality are characterized in terms of their extent, longevity and frequency. Impacts are communicated using a “traffic lights” metaphor for characterizing severity of water quality impairments arising from forestry and other anthropogenic pressures. The most serious impairments to water quality in managed boreal forests include (i) forestry activities causing excessive sediment mobilization and extirpation of aquatic species and (ii) other anthropogenic pressures caused by long-range transport of mercury and acidifying pollutants. The framework and tool presented here can help evaluate, summarize and communicate the most important issues in circumstances where land management and other anthropogenic pressures combine to impair water quality and may also assist in implementing the “polluter pays” principle.

## Introduction

Forests cover approximately 2/3 of Sweden and forestry contributes 2 % of GDP (Skogsstyrelsen 2014). Because they cover a relatively large proportion of the Baltic Sea drainage basin, runoff from Swedish forests has a major influence on water quality in the marine environment (Brandt et al. 2008). The vast majority of Swedish forests are managed for biomass production, and there are demands for further intensification to meet the goals of an emerging bioeconomy (Egnell et al. 2011). This near universal anthropogenic shaping of the forest landscape has been ongoing for several centuries, making it difficult to separate background or reference condition levels from the effects of present-day management activities (Renberg et al. 2009). Furthermore, Swedish forests have been subject to a range of non-forestry-related environmental stresses which have degraded water quality. Much of the forest area in southern Sweden is still recovering from the legacy of acid deposition (Akselsson et al. 2013; Moldan et al. 2013) which has led to ongoing surface water acidification (Futter et al. 2014) and slow biological recovery (Valinia et al. 2014). Most of the nitrogen (N) and mercury (Hg) deposited on Swedish forests is the result of emissions in other regions and long-range transport. Almost all of the organic micro pollutants (OMPs; including legacy and emerging persistent organic pollutants) are anthropogenic in origin. Forestry activities can, if carried out without proper consideration, exacerbate negative effects on water quality by altering rates of biogeochemical cycles, depleting element pools or mobilizing atmospherically deposited pollutants (Kreutzweiser et al. 2008; Lattimore et al. 2009; Laudon et al. 2011; Thiffault et al. 2011; Palviainen et al. 2015).

In 2000, member states in Europe adopted the Water Framework Directive (WFD) as an overall goal for water management (EC 2000). The WFD moved towards ecological integrity as a focal point of management instead of traditional sectoral strategies. This led to a comprehensive list of physical, biological and chemical parameters to be used when classifying surface waters in Europe (Hatton-Ellis 2008). The overall goal of the WFD is to reach Good Ecological Status (GES) which is defined as a state with minor influence from anthropogenic alterations, hence an undisturbed state (EC 2000, Annex V). The undisturbed state is determined by reference conditions, which are assumed to have existed before major industrialization, urbanization and intensification of agriculture (EC 2003a). The reference condition concept has been criticized for problems with interpretation and identification of the undisturbed state (Moss 2008; Hering et al. 2010; Valinia et al. 2012). In particular, it is important to recognize that reference conditions cannot and should not be equated with “natural conditions” (sensu Siipi 2008). Indeed, given the long history of human habitation and that almost all forests in Sweden are managed, reference conditions represent something of an idealization.

The WFD also enshrines the “polluter pays principle” (EC 2000) which embodies the concept that polluters are responsible for the pollution they have caused. While this principle appears simple, its implementation can be complicated, especially in situations where pollution is caused by more than one polluter (Lindhout and Van den Broek 2014). This is especially relevant in managed forests where water pollution may be the result of a combination of deposition of pollutants from long-range transport and their subsequent mobilization by forest management activities.

While water quality in managed Swedish forests is generally good when compared to agricultural and urban regions (Sponseller et al. 2014), as well as to other countries in Europe, there are valid concerns about the potential consequences of forestry activities for achieving Good Ecological Status. However, one of the main obstacles when using the WFD to communicate the effects of forestry on Swedish surface waters is that its complexity overwhelms foresters, decision makers, scientists and other actors (Futter et al. 2011; Berglund 2014; Keskitalo 2015) and that the results of status classifications can be counter-intuitive. For example, the “one out, all out” principle under which the worst result from a series of metrics (e.g. phytobenthos, fish and insects) is used for ecological status classification leads to near-pristine forest streams failing to achieve good ecological status (Löfgren et al. 2009). However, this could be resolved with type-specific reference conditions since using individual classification of surface water bodies as the WFD requires, where a naturally acidic system should be classified as naturally acidic without major anthropogenic influence. Compared to previous ecological quality criteria (EQC), where threshold values were used, naturally acidic systems would be wrongly classified.

Effects of forestry on boreal ecosystem status and surface water quality have been the subject of numerous reviews (Kreutzweiser et al. 2008; Bishop et al. 2009; Lattimore et al. 2009; Laudon et al. 2011; Thiffault et al. 2011; Palviainen et al. 2015). We have no intention of duplicating this material, but instead focus on frameworks for the conceptualization and communication of water quality issues related to forests and forestry.

We focus on eight surface water quality parameters which can be adversely affected by forestry or other anthropogenic activities. These include runoff volume, suspended sediments, N, phosphorus (P), dissolved organic carbon (DOC), base cations (BC; Calcium, Potassium, Sodium and Magnesium), Hg and OMPs. These parameters represent key physical and chemical attributes of streams, lakes and rivers; biological responses to anthropogenic disturbance are considered insofar as they are caused by the above eight issues. Hydromorphological alterations, while important, are not considered further.

Here, we propose a simple conceptual framework for evaluating biogeochemical cycles in the boreal forest and a tool for communicating the manner in which forestry operations may alter these cycles. We use the framework to explore controls on water quality in intact forests and to rank the impacts of forestry-related disturbances on water quality at local, landscape and national scales. Specifically, we pose three questions about water quality connected to forests and forest management. First, do forests or forestry affect the cycling of the chemical species in question, and if so, how strong is the effect?; second, what are the effects of present-day forestry on the water quality issue?; third, and most important, how certain is the science used to answer the first two questions?

## Conceptual frameworks

**Fig. 1:**
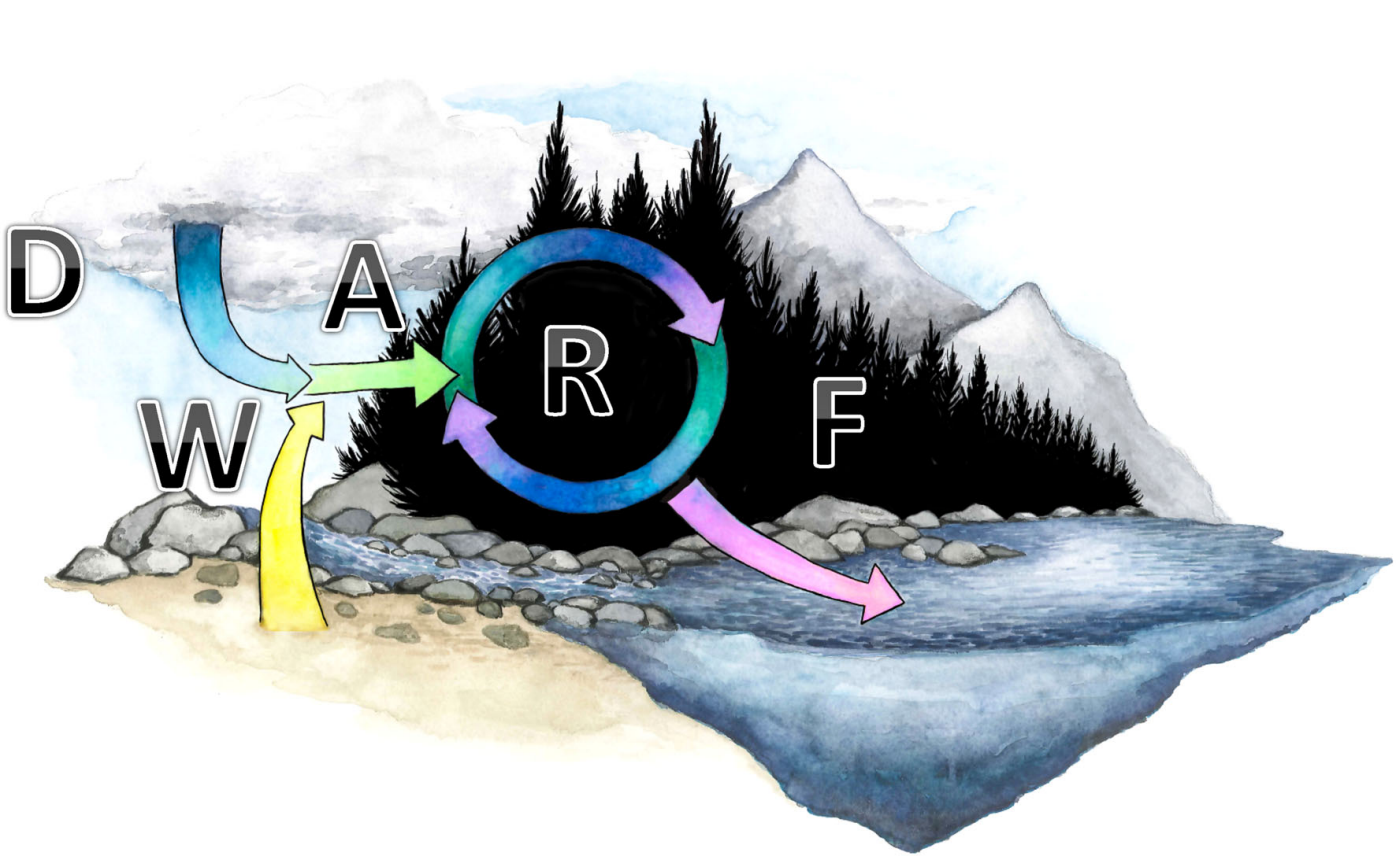
DWARF: a conceptual framework for forest biogeochemical cycles. Forest biogeochemical cycles are a combination of Deposition (D), Weathering (W), Accumulation (A) in soils or vegetation, Recirculation (R) between different stocks (i.e. vegetation, soils and litter) and Fluxes (F) to surface waters

Biogeochemical cycles in forest stands or headwater catchments can be conceptualized using the mnemonic DWARF: Deposition, Weathering, Accumulation, Recirculation and Flux (Fig. 1). *Deposition* is the wet or dry input of dissolved and particulate compounds and elements from the atmosphere to a forest stand. Deposition includes rain and snowfall. The “forest filter” effect and the waxy needles of conifers enhance the deposition of some classes of compounds, especially OMPs (Di Guardo et al. 2003; Nizzetto et al. 2006) and acidifying N and sulphur compounds (Helliwell et al. 2014). Typically, deposited pollutants are the product of long-range transport. *Weathering* is the physical, chemical or biological breakdown of geologic parent material. Weathering makes elements including phosphorus and base cations available for biological uptake and is the primary source of sediment. *Accumulation* is the process by which deposited and weathered materials are incorporated into the soil or biota. Accumulation also includes biological fixation of C and N from gaseous to organic form. Carbon fixation (i.e. photosynthesis) is the ultimate source of nearly all living and nonliving organic matter in forests, including DOC. *Recirculation* is a broad term which includes recycling, and redistribution of material within a stand. Examples of recycling processes include vertical transfers between plant and soil (as with litter fall and element uptake by roots), or the movement of base cations on and off ion exchange complexes. Redistributive processes include lateral redistribution of material within a stand including buildup of material in riparian zones and wetlands, paludification, the slow movement of contaminants through the soil profile and vertical redistribution related to e.g. podsolisation. *Fluxes* out of the system include gravity driven processes such as surface water runoff and mass wasting as well as the return of material to the atmosphere (e.g. via trace gas production or evapotranspiration). Redistribution of elements can be extremely important in delaying the impact of atmospheric deposition on stream water fluxes. Klaminder et al. (2011) suggested that the flux of atmospherically deposited lead in stream water might be delayed more than a century due to slow movement through the soil profile. There is also some evidence to suggest that changing rates of inputs can change outputs at a more rapid time scale. For example, Kothawala et al. (2011) showed that declines in atmospheric N deposition led to contemporaneous declines in stream water flux.

There can be positive feedbacks between the various components of the DWARF framework. For example, accumulation of material in growing forest biomass can enhance the “forest filter” effect whereby deposition of acidifying substances is increased. This, in turn, alters rates of base cation cycling (Helliwell et al. 2014). There is also some evidence from soil experiments that more rapidly growing forests (with higher assimilation rates) will affect base cation cycling through increased weathering rates (Palviainen et al. 2012).

Anthropogenic stressors, including forestry, alter the rates of one or more of the DWARF processes. These alterations may lead to impaired water quality, either through direct or indirect mechanisms. For example, atmospheric deposition of OMPs has a direct effect on their accumulation in forest ecosystems. On the other hand, acid deposition has an indirect effect on base cations, leading to alterations in their rates of weathering, accumulation and flux.

The magnitude of potentially negative effects of forest management on forest biogeochemical cycles can be conceptualized using the mnemonic ELF: extent, longevity and frequency. ELF can be used to weight the severity of forestry-related impacts to the spatial extent of an effect, its longevity and the frequency with which it occurs. For example, if it is assumed that elevated N leaching occurs after final felling of a whole stand (*E* = 1), for 10 years (*L* = 10) and a forest rotation lasts 100 years [thus *F* = 1/(rotation length)] then, at a stand scale, the ELF score is 1 × 10 × (1/100), or 0.1. It should be noted that the same ELF score will be obtained over a landscape where 0.01 of the stands are harvested on an annual frequency and the effect of individual harvesting events lasts 10 years.

$$ {\text{ELF }} = {\text{Extent}} \times {\text{Longevity}} \times {\text{Frequency}} $$

The downstream extent of negative effects is factored into the ELF score. For example, at a stand scale, E values greater than 1 will result if an impact is observed in both the stand and downstream watercourses. Quantifying the extent of downstream impacts can be somewhat subjective, especially when data are lacking. Forestry impacts that lead to significant negative effects downstream of harvested stands, including sediment pollution and Hg accumulation by fish in downstream lakes, will have higher ELF scores than impacts that are mostly observed at the stand level or immediately downstream (such as N leaching). The longer the duration of a negative effect, the higher the ELF score. If sediment pollution were to destroy the habitat of long-lived, slow-growing species such as freshwater pearl mussel (*Margaritifera margaritifera*) (Österling and Högberg 2014), it is possible that effect longevity would be greater than the length of a forest rotation. Such lags in the ecological effects of stream sedimentation have been observed elsewhere (Harding et al. 1998). Most negative effects are associated with final harvest. However, activities which occur more frequently, such as those caused by soil compaction associated with driving damage, will receive higher ELF scores.

**Fig. 2:**
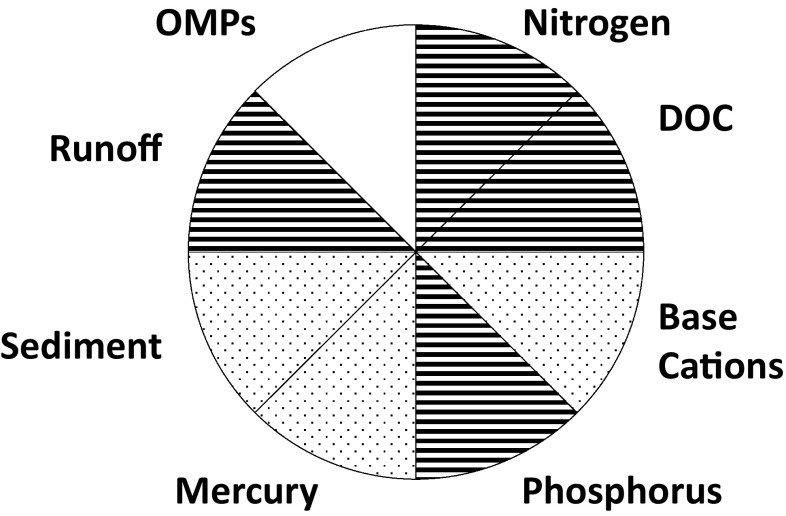
Pie chart showing ELF scores for forest water quality issues. Horizontal lines denote low ELF scores (i.e. <0.01) while dots are indicative of high scores (i.e. >0.01). There is insufficient information to assign values to blank cells

ELF scores are closely related to the scaling of water quality problems. Forestry activities that have a bigger footprint in space or time will typically have higher ELF scores. Regional and national scale problems are typically associated with spatially extensive or long-lasting forestry impacts. Because of the uncertainties associated primarily with longevity and assessing the extent of downstream influence, we report qualitative high and low values corresponding to ELF scores above and below 0.01, respectively. When sufficient data are not available to estimate an ELF score, as is the case with OMP, a value of “unknown” is reported (Fig. 2).

### Spatial scale

**Fig. 3:**
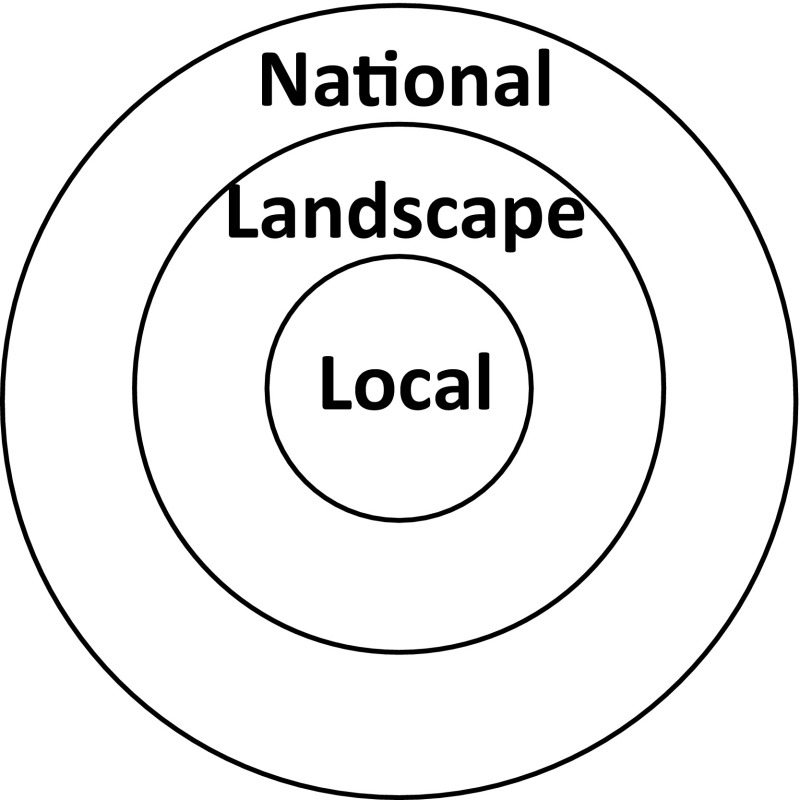
“Dart board” representation of spatial scales assessed here: local (headwater or stand scale effects) are presented in the innermost circle, landscape (10’s–100’s km^2^) scale effects are shown in the *middle circle* and national (Baltic Sea drainage basin) scale effects in the *outer circle*. Scale-dependent water quality impacts are communicated by overlaying the pie chart structure in Fig. 2 with the scale representation in this figure

Effects of anthropogenic activities including forestry on water quality can be manifested at local, landscape and national scales (Fig. 3). The local scale corresponds to individual forest stands or headwater catchments with areas of a few hectares to a maximum of approximately 10 km^2^. The landscape-scale is representative of tens to hundreds of km^2^. The national scale in our analysis is synonymous with the Baltic Sea drainage basin. The severity of each water quality issue and forestry effect is assessed at all three spatial scales. Effects at a local scale can be more or less severe at the landscape and national scales.

### Scale for issue severity, effect magnitude and uncertainty

**Fig. 4:**
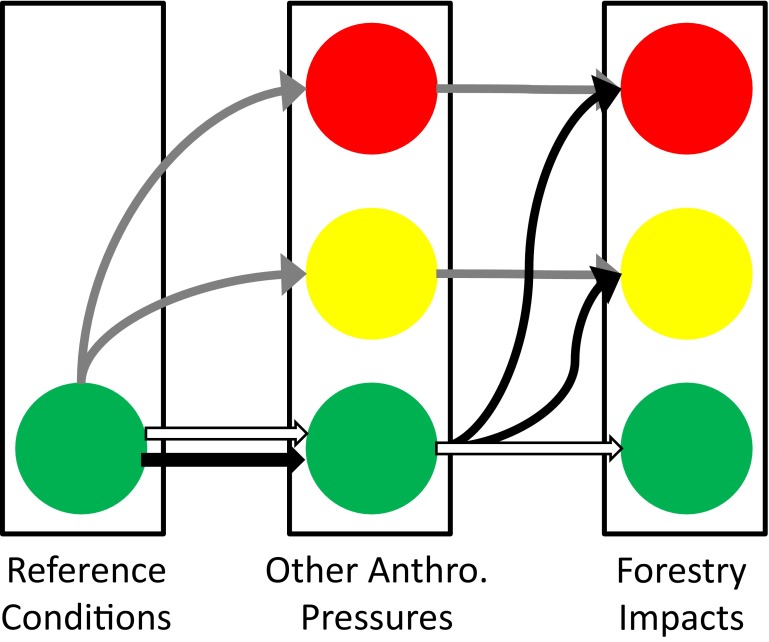
Possible trajectories in water quality as a result of impacts caused by other anthropogenic pressures or forestry. It is assumed that all surface waters are in reference conditions (*green*) when anthropogenic pressures are absent. Other anthropogenic pressures (e.g. long-range transport, climate change, etc.) may cause a range of deviations from reference conditions spanning from no (*green*) to moderate (*yellow*) severe impairments (*red*). Forestry may not lead to any further appreciable deviation in water quality above and beyond that caused by other anthropogenic pressures (*horizontal arrow*), or it may result in a further detectable deterioration of water quality. Type I trajectories are shown with *white arrows*; neither forestry nor other anthropogenic pressures lead to meaningful deviations from reference conditions. *Grey arrows* show Type II trajectories where other anthropogenic pressures lead to degraded water quality which is not further exacerbated by forestry. *Black arrows* show Type III trajectories where forestry is the cause of degraded water quality

**Fig. 5:**
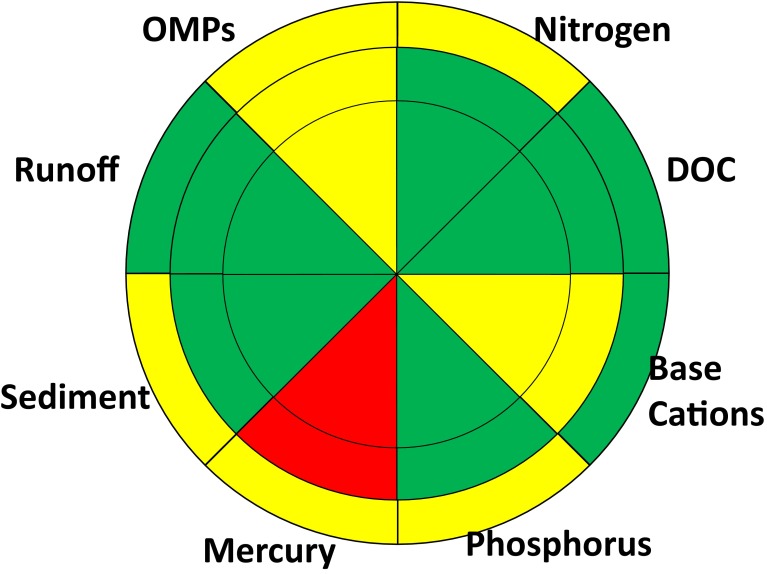
Water quality issues in the Swedish forest landscape at a local (*inner*), landscape (*middle*) and national (*outer*) scale caused by anthropogenic pressures other than forestry. Severity is coded as green (little or no impact), *yellow* (moderate impact) and *red* (severe impact) or *white* where there is too little information to make an assessment

**Fig. 6:**
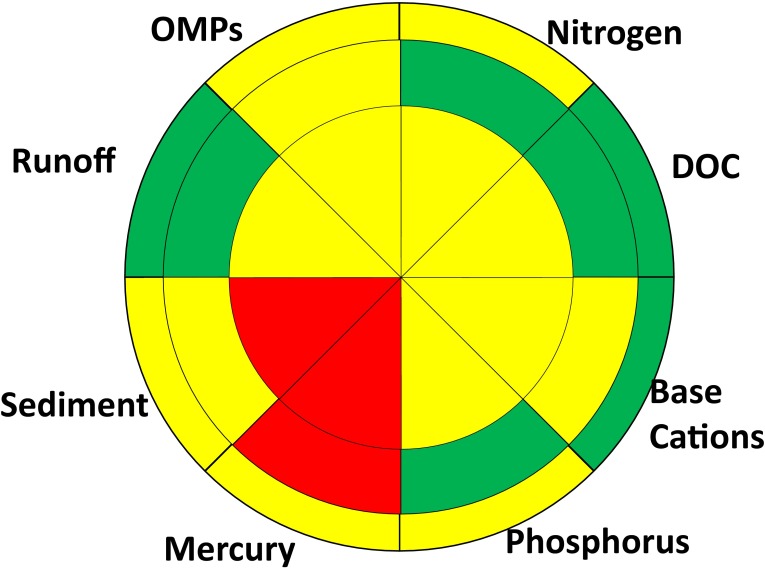
Net impacts of other anthropogenic pressures and forestry impacts on water quality in the forest landscape at local (*inner*), landscape (*middle*) and national (*outer circle*) scales. Severity is coded as green (little or no impact), *yellow* (moderate impact) and *red* (severe impact) or *white* where there is too little information to make an assessment

To visually summarize multiple water quality parameters, we use a “traffic light” coding of red, yellow and green to communicate severity and effect magnitude for different stressors (Figs. 4, 5, 6). When insufficient data are available to make an assessment, the cell is left blank. The “traffic light” approach has received widespread use in healthcare (Peters et al. 2007) and marine environmental assessment (Hargrave 2002; Foden et al. 2008). Foden et al. (2008) note that the strengths of the approach are that it provides users with a general, easy to track overview of impacts and gives a simplified presentation of potentially complex quantitative data. They caution, however, that any characterization scheme may be subjective and fine detail lost.

In the assessment presented here, minor impairments of water quality are coded green. A minor impairment is a detectable deviation from reference conditions which, on the basis of present scientific knowledge, is not believed to cause unacceptable harm to ecosystem function (i.e. minor impairments are analogous to WFD good ecological status). For instance, in acidification assessments of Swedish surface waters, the accepted deviation from reference conditions is a decline of 0.4 pH units since it is assumed that changes smaller than this do not adversely affect aquatic biota (Fölster et al. 2007).

Significant impairments of water quality are coded yellow. An impairment is deemed to be significant if it leads to an undesirable deviation from reference conditions. Conceptually, this deviation is analogous to the WFD moderate status (EC 2000, Annex V, WFD). Significant water quality impairments in the forest landscape can occur as a direct result of forestry activities or when other anthropogenic stressors have already pushed ecosystems into a degraded state. Thus, the relatively small impact of forestry on Baltic Sea eutrophication is still considered to be a significant impairment of water quality since that ecosystem is already in a degraded state due to excessive nutrient inputs from agriculture and sewage discharge. In a similar manner, forestry can potentially have a significant impact on base cation concentrations in surface waters already affected by acidification (Aherne et al. 2008; Zetterberg et al. 2013).

Severe impairments to water quality are coded red, similar to the WFD poor or bad ecological status (EC 2000; Annex V, WFD). An impairment is deemed to be severe if it results in unacceptable negative effects including demonstrable effect on human health, or if it leads to local- or regional-scale species extirpation. Severe impairments can be caused by both forestry activities and other anthropogenic pressures, primarily long-range atmospheric transport.

We assume that the effects of other anthropogenic pressures and forestry are additive. In the absence of forestry effects, other anthropogenic pressures such as long-range transport of pollutants cause one of the following: no appreciable deviation from reference conditions, significant, or severe impairments to water quality. Forestry may cause no further deterioration in water quality, or it may exacerbate the problem. The trajectories in Fig. 4 can thus be classified into three types depending on the traffic light colour under reference conditions, and due to other anthropogenic pressures and forestry.

- Type I—Neither forestry nor other anthropogenic impacts lead to appreciable deviations from reference conditions (white arrows in Fig. 4).
- Type II—Forestry does not appreciably worsen water quality above and beyond the effects of other anthropogenic pressures. That is to say, there is no increase in severity when moving from other anthropogenic pressures to forestry effects (grey arrows in Fig. 4).
- Type III—Forestry activities result in water quality impairments, whereas other anthropogenic pressures do not result in appreciable deviations from reference conditions (black arrows in Fig. 4).

It is possible that other anthropogenic pressures will cause significant impairments of water quality which are then exacerbated by forestry to cause severe degradation. However, none of the examples presented here appear to follow this trajectory.

While the framework presented here only accounts for negative effects of forestry on water quality, it should be noted that forestry can have positive effects, also. Globally, land use conversion through afforestation is widely used as a means of improving water quality (Neary et al. 2009) and can be an important contributor to sustainable flood management (Iacob et al. 2014). In Sweden, actively growing managed forests are strongly N retentive and thus may mitigate negative eutrophication and acidification effects in surface waters associated with excessive N deposition (Sponseller et al. 2016).

## Forests and forestry

Water quality in Swedish forests is directly and indirectly affected by a number of human activities. Emissions from fossil fuel burning in Sweden and elsewhere contribute to N pollution and exacerbate problems with base cations losses. In the past, forestry had a much greater impact on water quality than it does today. For example, alteration of river channels to facilitate log transport has had severe and long-lasting ecological consequences (Nilsson et al. 2005). While poorly planned forestry activities have the potential to negatively affect water quality, well-managed forests may have less water quality problems than some un-managed forests.

Here, we focus on stand-level forestry operations including site preparation, drainage, ash return, planting, thinning, fertilizing, fire prevention, final felling, harvesting and terrain transport. Thus, we do not consider water quality impairments associated with, *inter alia*, historical stream channel alteration for timber transport (Nilsson et al. 2005) or the negative effects of the forest products industry such as fibre banks associated with pulp mills on the Baltic coast (Assefa et al. 2014).

Severity of forestry impacts may differ depending on whether stem only (SOH) or whole tree (WTH) harvesting is practiced. Depending on site quality, the typical rotation time in a Swedish forest ranges between 60 and 120 years. Over that period, the following management activities are applied in the following, or slightly adjusted, sequence: final felling, biomass removal through harvesting, mechanical site preparation, optional ditch maintenance, planting, optional ash return, pre-commercial thinning, commercial thinning, optional fertilization and final felling. Throughout the rotation, road building and maintenance occurs. The majority of water quality impacts are associated with roads, harvesting (including thinning and final felling) and ditch maintenance.

**Fig. 7:**
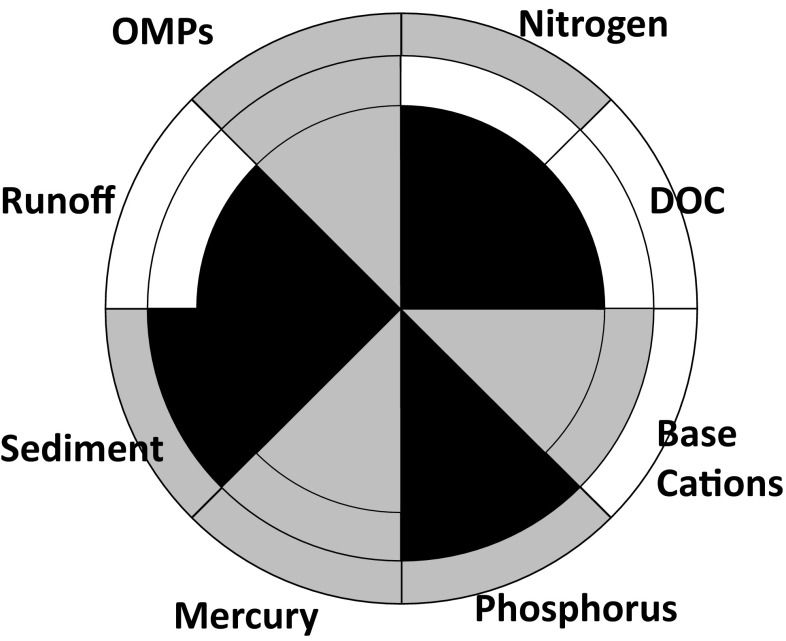
Impact type scores for water quality issues at local (*inner*), landscape (*middle*) and national (*outer circle*) scales. Type I impacts, shown in* white*, do not deviate significantly from reference conditions. Type II impacts, shown in* grey*, occur when other anthropogenic impacts are the primary reason for deterioration in water quality. Types III, shown in *black*, impacts occur when forestry is the primary cause of deterioration in water quality

The impacts of other anthropogenic stressors (Fig. 5) and their combined effects with forestry on water quality (Fig. 6) can be represented using the “traffic lights” colour coding from Fig. 4, the spatial scale representation (Fig. 3) and the water quality issue pie chart (Fig. 2). The differences in colours between the other anthropogenic stressor effects (Fig. 5) and combined impacts (Fig. 6) are related to impact type trajectories (Fig. 4). The impact types for different spatial scales and water quality issues are graphically summarized in Fig. 7. It is notable that forestry is not responsible for any Baltic-scale effects and a minority of landscape-scale impacts (Fig. 7).

### Runoff

The hydrological cycle is the key driver of forest biogeochemical cycling. Globally, precipitation is a limiting factor for forest establishment in many regions. In the boreal ecozone, water is generally not the primary limiting factor for forest growth, and a significant fraction of annual precipitation falls as snow. The effects of forests and forestry on the hydrological cycle are strongly scale dependent (Ellison et al. 2012). Intact forests return a significant fraction of incoming precipitation to the atmosphere through evaporation and transpiration and afforestation can effectively reduce runoff (Iacob et al. 2014). Felling reduces transpiration and canopy interception, leading to wetter soils, a greater fraction of precipitation contributing to runoff, and increased lateral fluxes of water (for a recent review of the processes, see Launiainen et al. 2014). Wetter soils can contribute to increases in surface water DOC concentration (Schelker et al. 2013), mercury methylation rates (Lattimore et al. 2009) and potentially production of greenhouse gases (e.g. CH_4_ and NO_2_) from anoxic soils (Vor et al. 2003). Forest ditches which are established to dry out soils so as to improve forest growth in saturated areas increase water fluxes beyond reference condition levels and their maintenance can lead to elevated fluxes of sediments and nutrients (Manninen 1998). Forestry operations on wet soils are an underappreciated threat to water quality; Laudon et al. (2016) discuss some of the issues of soil wetness and consequences for forests and forestry. At a local scale, forestry has a Type III effect on runoff.

### Nitrogen

Nitrogen is an essential plant nutrient (see Sponseller et al. 2016) that also can limit rates of biological processes in boreal streams (Burrows et al. 2015) and lakes (Bergström et al. 2008). Atmospheric N deposition has increased considerably over the past 100 years as a result of fossil fuel burning and increased fertilizer use. The health of the Baltic Sea ecosystem is under threat from excessive N inputs associated mostly with sewage and agriculture (Conley 2012). Tree growth in most Swedish forests is N-limited and N fertilizer is added into approximately 25 000 ha annually in northern and central Sweden to increase yields. Over the course of a whole rotation, boreal forests tend to be net N sinks, in that they effectively take up the N deposition derived from fossil fuel burning in Sweden and elsewhere.

Forestry activities affect the accumulation, recirculation and fluxes of N from forest stands. SOH and WTH remove N from the stand, decreasing the size of the N pool and potentially slowing rates of recirculation (Lundborg 1997; Palviainen and Finér 2012). Effects are more pronounced with WTH due to the removal of large amounts of N in needles. While N leakage can occur following final felling, the total amount lost is small relative to total atmospheric deposition (Futter et al. 2010). The concentrations of N in groundwater following final felling are elevated when compared to undisturbed forests but are not high enough to cause problems of compliance with European legislation or human health issues. However, forest lands are the largest single-net source of N entering the Baltic Sea from Sweden (Brandt et al. 2008). Forestry clearly causes local increases in N fluxes but the legacy of greater deposition and other pollution sources in the Baltic Sea catchment mean that many of the negative impacts are caused by other anthropogenic pressures. Forestry has a Type III effect on N at the local scale; the legacy of atmospheric deposition contributes to the Type II effect at the landscape and national scales.

### Phosphorus

Phosphorus (P) is also an essential plant nutrient that can further influence algal growth in lakes and rivers. This effect is most pronounced in southern Sweden, where significant atmospheric deposition means that systems are not N-limited. Very little P is lost from intact forests. However, significant amounts can be released when soils or sediments are disturbed during site preparation and ditch clearing. At the local scale, levels of P in surface waters can be high enough to cause significant changes in aquatic plant communities.

Following harvest, P is removed in biomass. Ditch maintenance may increase P fluxes out of the affected stands through the mobilization of sediments (Manninen 1998). Increases in both concentration and flux of particulate phosphorus may be seen even when soil disturbance is minimal as the increased runoff following clearfelling can flush fine sediments from ditches (Kaila et al. 2014). The local scale effects of forestry operations on P cycling must be balanced against the observed long-term decline in tree mineral nutrition status and the increasing likelihood that forests are P limited (Jonard et al. 2015). At a national scale, the situation for P is similar to that for N. Any additional inputs are problematic for the already eutrophied Baltic Sea ecosystem. At local and national scales, forestry results in Type III effects on surface water P.

### Base cations

Base cations (Calcium, Magnesium, Potassium and Sodium) are essential plant nutrients and some of the most important elements buffering soil and surface water acidification. Acid deposition increases the rate at which base cations are leached from the soil. Following reductions in acid deposition, surface water base cation concentrations may decline further due to lack of a mobile co-anion for transport. The acidification caused by long-range pollutant transport is largely an issue of the past. However, modelling studies have suggested that whole tree harvesting, which may remove base cations from forest soils (Zetterberg et al. 2013) faster than they can be replaced by mineral weathering. If this were to occur, it could possibly lead to further acidification of sensitive waters (Akselsson et al. 2007). Unfortunately, weathering rates are too uncertain to draw firm conclusions about the sustainability of forest harvesting (Klaminder et al. 2011; Futter et al. 2012). However, experiments suggest that more rapidly growing forests may increase weathering rates (Palviainen et al. 2012).

Water quality impairment associated with declining base cation concentrations probably follows a Type II trajectory. The regional legacy of acid deposition has depleted soil base cations, resulting in ongoing acidification of many soils (Akselsson et al. 2013) and surface waters (Moldan et al. 2013; Futter et al. 2014) in southern Sweden. Biomass removal following forest harvest will reduce the BC pool in a stand, leading to reductions in the rates of recirculation and potentially lower fluxes to surface waters.

### Dissolved organic carbon

Dissolved organic carbon (DOC) originates ultimately from plants fixing atmospheric carbon and is derived from the breakdown of plant material in soils and litter. Concentrations of DOC are increasing in many surface waters and it has been hypothesized that declines in acid deposition (Monteith et al. 2007; Valinia et al. 2015), historical land management practices (Meyer-Jacob et al. 2015) and a changing climate (Oni et al. 2014) are important drivers. This is a concern for a number of reasons. DOC is a naturally occurring acid that if elevated above its reference condition can contribute to a delay in acidification recovery (Futter et al. 2014) and acidity-related fish kills in some parts of Sweden (Serrano et al. 2008). Elevated DOC concentrations can lead to significant alterations of lake ecology including changing the light environment which inhibits gross primary productivity (Solomon et al. 2015), fuelling heterotrophic processes and altering the amount and bioavailability of contaminants (Rask et al. 2014). Finally, the flux of DOC from Swedish forests may contribute to acidification in the Baltic Sea (Omstedt et al. 2010).

Final harvesting almost always leads to increases in surface water DOC (e.g. Schelker et al. 2012; Palviainen et al. 2015). This effect is difficult to detect at all but the smallest spatial scales (Lepistö et al. 2014). Thus, forestry has a Type III effect on DOC at the local scale.

### Mercury

Mercury (Hg) is a potent neurotoxin which is banned in Sweden. There is a high degree of concern about mercury in Swedish forest waters (Eklöf et al. 2016). In its methylated form (MeHg), it is able to bio-accumulate in food webs and cause neurological damage in humans, other mammals and birds. Concentrations of MeHg in fish from many Swedish lakes are high enough to constitute a possible human health risk (Åkerblom et al. 2014). The environmental behaviour of mercury is complicated: MeHg is produced in environments with low-ambient oxygen concentrations, including lake sediments and wetlands with high concentrations of DOC. Forestry activities (such as final felling) which result in wetter soils in some cases can lead to higher concentrations of MeHg (Porvari et al. 2003), which in turn can result in elevated Hg concentrations in fish (Garcia and Carignan 2005; Martin 2014). In a survey of intact and harvested Swedish sites, Skyllberg et al. (2009) observed significantly higher MeHg concentrations in streams draining areas with clearcuts than those draining intact forests. However, de Wit et al. (2014) reported no increase in MeHg concentrations following clearcutting in a Norwegian study.

The trajectories for Hg are assigned to Type II at all spatial scales. While it is clear that forestry activities can sometimes lead to increased Hg fluxes, the Hg concentrations and fluxes associated with the legacy of atmospheric deposition will continue to pose health threats for many years to come even in the absence of forestry.

### Organic micro pollutants

Organic micro pollutants (OMPs) include a wide range of natural and anthropogenic compounds including dioxins, polychlorinated biphenyls (PCBs), polycyclic aromatic hydrocarbons (PAHs) and perfluorinated compounds (PFAs). Many of these compounds are found at toxic levels in Baltic Sea biota and sediments. With few exceptions, OMP accumulate in boreal forests as a result of wet and dry atmospheric deposition. While their concentrations are typically very low in Swedish forests, they are a potential concern because, if mobilized, they can be transported to the Baltic Sea. Very little is known about the behaviour of OMPs in Swedish forests. They appear to be co-transported with organic carbon (Bergknut et al. 2011a) and can be found at concentrations similar to those in contaminated sites (Bergknut et al. 2011b). There are large uncertainties in estimates of OMP fluxes and further research is needed to evaluate the importance of boreal forest waters as a source of OMPs to the Baltic Sea. It is not clear what effect forestry operations will have on OMP cycling, but it is likely that activities which contribute to increased fluxes of DOC and sediments will also increase flux of OMP. This is of special concern at the national scale as any extra inputs of OMPs to the Baltic Sea are undesirable. Because of the uncertainty associated with forestry effects on OMP cycling and the clear link between long-range transport and subsequent deposition, this issue is coded as Type II at all spatial scales.

### Sediments

Sediments can be mobilized as a result of increased runoff following final felling, ditch maintenance and site preparation. Excess suspended sediments can have serious negative effects on aquatic biota (Wood and Armitage 1997). The sediments produced by ditch clearing and poorly planned or constructed forest roads and stream crossings can be a serious water quality issue. Forestry activities can have both direct and indirect effects on water quality. There are direct negative effects of increased sediment loads on aquatic habitat (Stenberg et al. 2015) as well as indirect effects associated with co-transport of nutrients and contaminants. Specifically, sediments can transport and subsequently release large amounts of P (Kaila et al. 2014). More importantly, sediments can destroy aquatic habitats, smother spawning beds, cause the loss of fish populations, and severely alter the abundance and biodiversity of aquatic invertebrates (Burdon et al. 2013). While sediment pollution is often a local issue, the effects can be long-lasting as it can take many years for habitats to recover and be re-colonized (Harding et al. 1998). For example, if excess sediment results in extirpation of freshwater pearl mussels, it can take decades before recolonization occurs (Österling et al. 2010). Kreutzweiser et al. (2009) suggest that environmentally sensitive forestry practices, which take extra precautions when working near water, can potentially minimize sediment pollution. While most local-scale impacts are the result of too much sediment, too little sediment can also be problematic. Legacy hydromorphological alterations to river channels to facilitate log transport led to lowered sediment production and transport. At the national scale, hydroelectricity reservoir impoundments have resulted in declines in sediment transport with negative effects on Baltic Sea silica concentrations (Humborg et al. 2000). Thus, sediments are coded as Type III at the local and landscape-scale but Type II at the national scale.

### Uncertainty

**Fig. 8:**
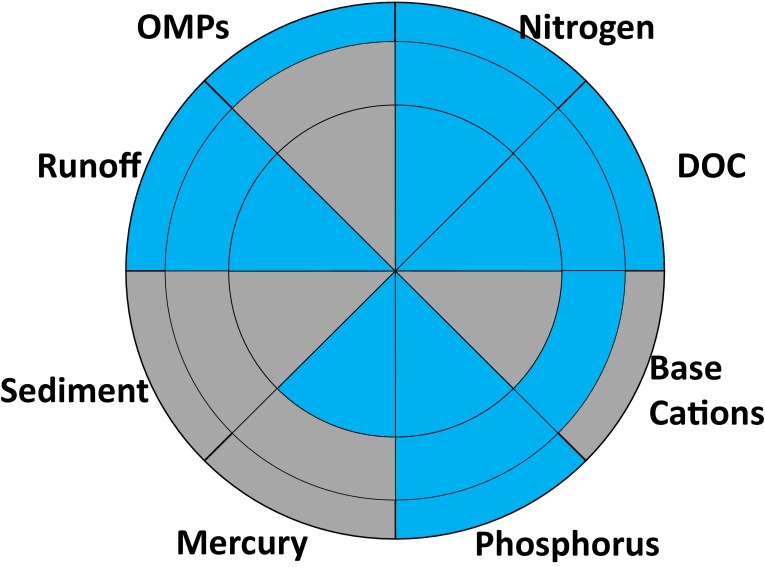
Uncertainty associated with water quality issues and forestry impacts at local (*inner*), landscape (*middle*) and national (*outer circle*) scales. Cells are coded *blue* when there is limited or no uncertainty and *grey* where there is significant uncertainty associated with forestry impacts on a water quality issue at the local (*inner circle*), landscape (*middle circle*) or national (*outer circle*) scales

There is some degree of scientific uncertainty about all environmental issues and there are significant challenges in communicating this to decision makers (Beven 2010; Petersen 2012). The science behind forest water quality and forestry related impacts is more or less certain, depending on the particular issue. We have used sky colours to represent, and what, in our opinion, is the relevant degree of uncertainty with each issue (Fig. 8). Under blue skies, it is possible to obtain a relatively good view of the surrounding landscape. When skies are grey as a result of low clouds or fog, features in the surrounding landscape are less certain. Thus, a high degree of certainty is coloured blue and a low degree of certainty is coloured grey.

Effects of forestry on N, P and DOC and runoff in the boreal forest are relatively well understood at all spatial scales. The potential local scale effects of forest harvesting on Hg cycling are also well documented (Bishop et al. 2009; Eklöf et al. 2016), but more work is needed to upscale these results to regional and Baltic Sea levels. The effects of forestry on base cation cycling are uncertain and need further investigation as modelling and measurement suggest contradictory results. The water quality impacts of OMP are not well established at either the stand or landscape level. However, it is clear that any additional loading of these compounds to the Baltic is undesirable. The most important knowledge gaps are related to sediment production and mobilization. It seems highly likely that excessive sediment mobilization is having widespread negative effects on aquatic biota dependent on well-oxygenated streambeds.

## Discussion

Successful policy implementation is dependent on a dialogue between all relevant stakeholders, as their involvement leads to a diversity of experiences and views and knowledge (EC 2003b). The framework presented here can help this dialogue as it provides a set of tools for communicating the potential effects of forestry and other sources of impaired water quality to policy makers, regulators, land managers and other stakeholders. It provides a “dashboard” for the forestry sector and decision makers to quantify, assess and communicate water quality-related risks associated with forestry activities on a level that is understandable. This may be especially helpful in linking top-down and bottom-up initiatives to maintain or improve forest water quality.

The connection between WFD measures for achieving good ecological status and Swedish forestry is quite weak, without any real guidance about programs of measures to improve water quality (Futter et al. 2012; Berglund 2014). As it is today, the WFD mandates ecological status assessment on the basis of deviations from reference conditions. As shown in Fig. 8, there are significant uncertainties associated with forestry effects on water quality. The uncertainty in reference condition estimates reduces the credibility of water management systems and complicates communication with stakeholders in the forest sector. Furthermore, the relatively short-5-year planning cycles in the WFD may be inappropriate for forest management based on a whole rotation. It has been suggested that 100-year planning cycles would be more appropriate in the WFD (Josefsson 2012). This would be more consistent with the 60–120-year rotation period used in forest planning.

The WFD enshrines the “polluter pays principle”, the goal of which is to ensure that those who cause water pollution are held responsible for pollution monitoring and cleanup (Lindhout and Van den Broek 2014). Today, it is easy for relevant authorities to identify point source polluters, while sectors such as forestry and other recipients of long-range transported pollutants pose challenges in application of the polluter pays principle. While it is clear that the forestry sector should be held accountable for the direct impacts of forestry related water pollution, the responsibility of actors in the forestry sector for water quality impairments caused by other anthropogenic actions is less clear. While forestry operations should be as environmentally sensitive as possible, it does not seem entirely appropriate to hold the forest industry responsible for the legacy of impaired water quality caused by long-range pollutant transport. Forestry measures to maintain or improve water quality should focus on Type III issues where forest management is the main cause of water quality impairments. Remediation of Type II water quality issues caused primarily by other anthropogenic pressures cannot be the sole responsibility of the forestry sector.

Top-down, regulatory approaches to water quality management must be complemented by non-policy options such as forest certification (Lattimore et al. 2009) and bottom-up initiatives. For example, Nordlund et al. (2014) report on attitudes of forest machine operators to soil disturbance associated with driving damage. In general, machine operators were sensitive to and aware of the potential for driving damage and water quality impairment. The results of the framework analysis presented here, showing the potentially severe negative consequences of forestry activities on sediment mobilization could help to reinforce the sense of stewardship already felt by some actors in the forest sector. Specifically, forestry operations should be conducted in a manner which minimize sediment loads to surface waters. This could include hydro-mapping measures (Laudon et al. 2016) such as water sensitive driving, better road planning and use of brash to minimize soil compression.

Furthermore, separating the effects of forestry from other anthropogenic stressors could help to achieve more ethical forest management. Berglund (2014) notes that participatory approaches are needed in forest management. The conceptual framework presented here can be used as a simple tool to facilitate dialogue between the forestry sector, relevant authorities and other stakeholders so as to achieve a deliberative democracy and work towards consensually agreed upon goals as prescribed by the WFD. We believe that this framework could aid in the democratic process by allowing all stakeholders to rank and communicate the effects a management decision may have on forest surface waters. Newig et al. (2005) have stated that public participation is a key component for reducing uncertainties in the WFD planning and implementation process. This framework will encourage participation from local to national levels and present the effects of forestry while at the same time facilitating active involvement from stakeholders. Furthermore, the simplicity of this approach offers the possibility to use the conceptual framework outside Europe and for sectors other than forestry.

## Conclusions

Water quality in Swedish forests is generally good, and the effects of modern forestry are often relatively minor when compared to other industries and to past forestry activities. This does not mean we can be complacent. Any forestry activity leading to increased sediment mobilization can have serious negative consequences and the legacy of OMP and Hg deposition is a persistent and pernicious threat to water quality, whether forestry occurs or not. Also, any activity which results in increased nutrient fluxes to the Baltic is a concern. Climate change and increasing demands for bio-energy may alter forest management strategies, leading to more N, P, Hg and sediment pollution. Lastly, overcoming the legacy of forest ditching may be difficult or impossible. However, the simple conceptual framework presented here creates an opportunity for relevant authorities, actors and other stakeholders to identify, rank and communicate potential effects of forestry at local, regional and national scales. It also gives the forestry sector the opportunity to measure its effects (direct and indirect) against long-range pollution. By identifying those responsible for impaired water quality, appropriate measures for enforcing the polluter pays principle can be developed and appropriate remediation measures can be taken.
